# The rediscovery of the Great Winterberg endemic *Lotononis harveyi* B.–E.van Wyk after 147 years, and notes on the poorly known Amathole endemic *Macowania revoluta* Oliv. (southern Great Escarpment, South Africa)

**DOI:** 10.3897/phytokeys.62.8348

**Published:** 2016-04-15

**Authors:** Vincent Ralph Clark, Joanne Bentley, Anthony P. Dold, Vathiswa Zikishe, Nigel P. Barker

**Affiliations:** 1 Great Escarpment Biodiversity Programme, Department of Botany, Rhodes University, Grahamstown, 6140, South Africa; 2 Department of Molecular & Cell Biology, University of Cape Town, Rondebosch, 7700, South Africa; 3 Selmar Schonland Herbarium, Department of Botany, Rhodes University, Grahamstown, 6140, South Africa; 4 South African National Biodiversity Institute, Threatened Plants Programme, c/o Department of Botany, Rhodes University, Grahamstown, 6140, South Africa; 5 Great Escarpment Biodiversity Programme, School of Plant & Crops Sciences, University of Pretoria, Hatfield, 0028, South Africa

**Keywords:** Lotononis harveyi, Macowania revoluta, Great Winterberg, Amatholes, endemic, rediscovery, fieldwork, Red Data status, Great Escarpment, South Africa, Eastern Cape

## Abstract

South Africa’s 800 km-long southern Great Escarpment hosts numerous endemic plant species only known from their type specimens or from very few records. This is a legacy of a 100–150 year lag between the pioneer work of 19^th^ century botanists and repeat fieldwork in the 21^st^ century. As a result, population and ecological data are lacking for many local endemic species. Here we report on the rediscovery of *Lotononis harveyi* B.–E.van Wyk 147 years after its original description, and provide the first detailed ecological notes on the poorly known shrub *Macowania revoluta* Oliv. Both species are locally endemic to the Great Winterberg–Amatholes (Eastern Cape Province). With only six known individuals, *Lotononis harveyi* is recommended the conservation status of Critically Endangered, with fire (and potentially grazing) being the main population constraints. *Macowania revoluta* is locally abundant, and it is surprising that it has been so poorly collected in recent decades. It occupies an important local niche as a keystone montane wetland species, and its narrow distribution range – combined with pressure from woody alien invasive species – suggests that its conservation status should be Rare. The research further highlights the need for continued biodiversity field research along South Africa’s poorly explored Great Escarpment.

## Introduction

The ‘Cape Midlands Escarpment’ (comprising the Sneeuberg, Great Winterberg–Amatholes (GWA) and Stormberg, mostly in the Eastern Cape Province, South Africa) has been part of a southern Great Escarpment biodiversity research focus since 2005 (Clark 2010, Clark et al. 2009, 2014). Despite the numerous rediscoveries and species new to science (Goldblatt and Manning 2007, Nordenstam et al. 2009, Stirton et al. 2012, Boatwright and Manning 2013, Rourke et al. 2014, Clark et al. 2015), several endemic plant species only known from their type specimens have remained elusive, and the ecology of several others is still very poorly known. For instance, Clark et al. (2014) indicate that eight (23%) of the c.36 plant species endemic to the GWA are still only known from their type specimens.

Here we provide detailed notes on two of these poorly-known GWA endemics: *Lotononis harveyi* B.–E.van Wyk, rediscovered 147 years after its description in *Flora Capensis*, and first-time population and ecological data for *Macowania revoluta* Oliv., last reliably collected some 40 years ago.

## Systematic

### Lotononis harveyi

Taxon classificationPlantaeFabalesFabaceae

B.–E.van Wyk

Fig. 1 ; Plate 1

#### Remarks.

Described by William Harvey in *Flora Capensis* as *Buchenroedera spicata* Harv. in 1862 (Harvey and Sonder 1862), this species was collected (without date) by Mrs Elizabeth Mary Barber sometime in the 1800s on the ‘Winterberg’. Three vouchers of this original material exist: one in the Bolus Herbarium (BOL), under her own initials; one in Kew (K) under F.W. Barber, her husband’s initials; and one in Trinity College Dublin Herbarium (TCD), also under her own initials. At the time of Van Wyk’s (1991) ‘*Synopsis of the genus* Lotononis’, this species was still only known from the type material. This remained the case when Clark et al. (2014) published their overview of plant diversity and endemism in the GWA.

Extensive fieldwork by VRC in the Great Winterberg in January 2009 for his PhD resulted in the first recollection of this species since its publication in *Flora Capensis*, although this was not realised at the time. The specimen (*Clark VR, Pienaar C, Daniels R 316*; GRA, NBG) was given the tentative identification Lotononis cf. viminea (E.Mey.) B.-E.van Wyk until re-examination in 2014 suggested that it was in fact *Lotononis harveyi*. A follow-up expedition to find more plants was undertaken on the 6^th^ November 2014, based on the 2009 specimen having been in fruit in January 2009, as it was thought the plants might flower in November–December.

The 2009 site (hereafter Locality 1) was relocated without difficulty, and the search extended southwards down the 19^th^ century wagon trail to the trigonomic beacon and eastwards to the edge of Paradise Kloof (part of the Fenella Falls gorge complex), covering approximately one square kilometre. Despite exploring the area carefully (in the plateau grassland and along the edge of the ravine, as well as in the fynbos and grassland on the steep slopes of the ravine) only six individual plants were found (three in flower, three not).

**Plate 1.**
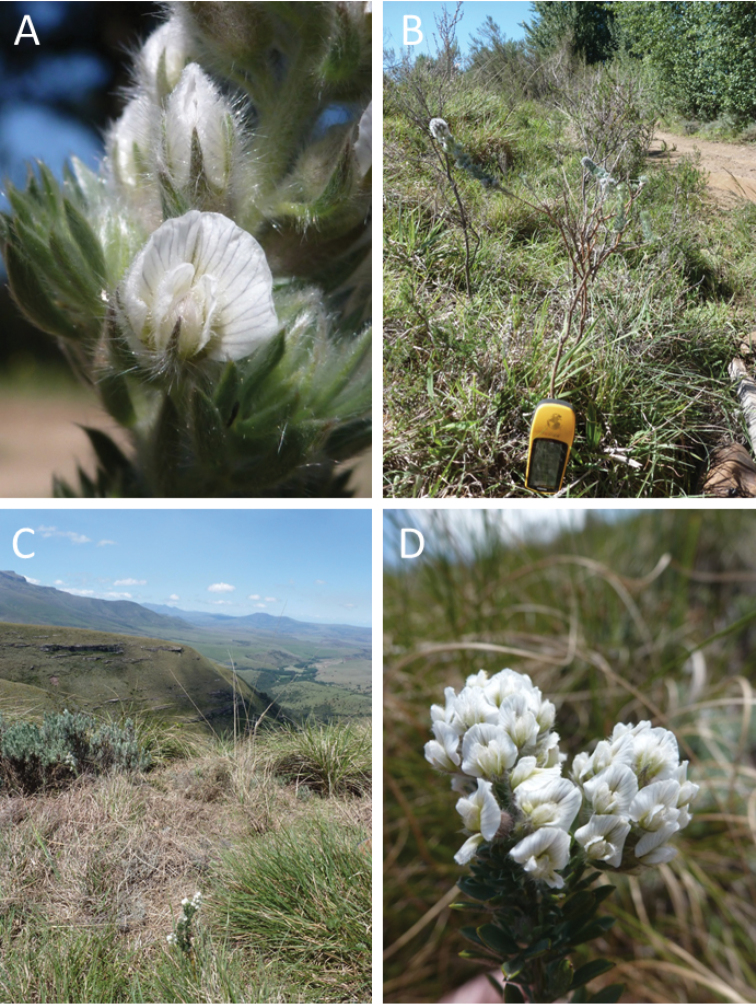
The first ever photographs of the Great Winterberg–Amatholes endemic *Lotononis harveyi* B.–E.van Wyk **A** flower detail (*Clark VR, Bentley L 9*) **B** habit (*Clark VR, Bentley L 9*; the Garmin GPS indicates scale) **C** montane grassland habitat, with *Lotononis harveyi* in the middle foreground (*Clark VR, Bentley L 11*) **D** complete and open inflorescences (*Clark VR, Bentley L 12*). Photographs by V.R. Clark.

#### Key characters confirming rediscovery.

The 2009 specimen was assigned to *Lotononis harveyi* on the basis of the elongated racemes (therefore not *Lotononis trichodes* (E.Mey.) B.-E.van Wyk, another local Great Winterberg endemic); the white flowers with densely hairy petals (based on the label information and a few remaining petals on the specimen, as the plant was mostly in pod); the long calyx lobes, hirsute leaves, and long stipules (which match Van Wyk’s 1991 figure 89 very well). The identification was confirmed by the November 2014 plants, especially by the white, hairy petals.

#### Population assessment.

The plants at the three localities are described separately:

In 2009, two plants were found and collected at Locality 1, recorded as ca. 50 cm tall and with white flowers. This site occupies two square meters and is located on the two meter-wide ‘middle man’ between the 19^th^ century wagon trail over the Great Winterberg and the current Finella Falls farm access road. In November 2014, at the same site, three plants were found. One was 45–50 cm tall, branched and in full flower. The other two were 15 cm and 5 cm tall respectively, both damaged on their main axes (probably being the two specimens collected in 2009, one lodged in the Selmar Schonland Herbarium, GRA, and a duplicate to the Compton Herbarium, NBG) but shooting side branches; neither were flowering. Locality 2, situated on the western lip of Paradise Kloof, comprised one plant 15 cm tall, in flower. Locality 3, only a little further back from Locality 2, contained two plants: one 30 cm tall, in flower, the other 20 cm tall, not in flower.

#### Habit and ecology.

Van Wyk (1991) indicated that the habit of *Lotononis harveyi* was not known. From the recent collections it can now be stated that it is an erect to spreading woody shrublet 20–50 cm tall, comparing well with Mrs Barber’s notes on her TCD voucher: ‘*about a foot and a half high – slender with very few branches – perennial*’. As was postulated by Van Wyk (1991), it is indeed distinct from the prostrate habit of *Lotononis trichodes*. Mrs Barber notes on her TCD voucher ‘*blossoms in autumn*’, and as we found the species in full bloom in November, *Lotononis harveyi* perhaps flowers in sync with the bimodal rainfall regime dominant in this area, i.e. early and late summer (Mucina and Rutherford 2006).

If the two smaller plants recorded in November 2014 at Locality 1 are indeed the survivors of the two 2009 vouchers, their limited growth since then suggests that the species grows extremely slowly, and this may partly account for its apparent rarity. In contrast, it is surprising that there is no obvious evidence of recruitment despite the floribund inflorescences.

#### Habitat.

Generally speaking, *Lotononis harveyi* occurs in Amathole Montane Grassland (Mucina and Rutherford 2006). The habitat conditions at each locality are discussed separately to identify common ecological factors which may account for this species’ apparent rarity.

Locality 1 consists of a very small area of moribund *Themeda triandra* Forssk. grassland. Other species present in this area are *Cliffortia* sp. (50–60 cm tall), *Luzula africana* Drège ex Steud. and *Fingerhuthia sesleriiformis* Nees. The remainder of the road reserve comprises the invasive tree Populus × canescens (Aiton) Sm.. The soil is deep and clayey. No plants were evident in the grassland on either side of the road reserve: these grasslands comprise well-gazed *Themeda triandra* grassland studded with tall *Festuca costata* Nees tussocks. The gradients are gentle, soils deep and rich, there is limited rockiness, and the grass is probably burnt on a regular basis to limit the spread of the unpalatable *Festuca costata*.

Locality 2 comprised (prior to burning) *Tenaxia disticha* (Nees) N.P.Barker & H.P.Linder (=*Merxmuellera disticha* (Nees) Conert)–*Themeda triandra*–*Festuca costata* grassland with the fynbos elements *Erica leucopelta* Tausch, *Searsia rosmarinifolia* (Vahl) F.A.Barkley, and shrubs/trailers such as Rubus ludwigii Eckl. & Zeyh. subsp. ludwigii and *Rubus rigidus* Sm.. The edge of the plateau comprises rocky sandstone outcrops favoured by the fynbos elements, while away from this the soil is a deeper, loamy clay.

Locality 3 comprises moribund *Tenaxia disticha*–*Themeda triandra*–*Festuca costata* veld with scattered *Arrowsmithia styphelioides* DC. dwarf shrublets and *Helichrysum splendidum* (Thunb.) Less.. Fire has evidently been absent for some time.

Mrs Barber’s TCD voucher notes that her specimens grew ‘*amongst the rocks and long grass*’ and in ‘*good soil*’. This – together with the six plants all being found in fire-exclusion areas or moribund grassland – suggests that the species is susceptible to fire and possibly grazing pressure. There is no currently no indication on whether this species is a resprouter or a reseeder, and research into the autecology of this species is warranted.

#### Conservation status and threats.

*Lotononis harveyi* is currently listed as Data Deficient (Victor and Dold 2005). Based on our observations we suggest that it be considered ‘Critically Endangered’ until more surveys in the general area are carried out. Currently virtually nothing about its biology is known, and accordingly no concrete conservation recommendations can be made. Possible general threats are the over-use of fire (a fire management history of the relevant farms can probably be obtained to indicate fire frequency), although fire has been a natural part of the ecology of these mountains well prior to the discovery of this species.

The general area is vulnerable to invasion by *Rosa rubiginosa* L. (a fast-emerging invader, with several seen in Localities 1 & 3) and *Pinus patula* Schltdl. & Cham. (Locality 2), while Locality 1 is in danger of being overrun by Populus × canescens. The targeting of mountain environments for wind farms in South Africa is another concern, with potentially detrimental impacts on localised endemics such as *Lotononis harveyi*.

#### Areas for further exploration.

A more exhaustive search along the rugged, extensive rocky rims of the Fenella Gorge area and perhaps on the (still unexplored) slopes of Mount Frederick and Besterskop (the promontory below the main Great Winterberg peak) and the scarp slopes below The Ruitjies might produce more plants. In fact, much of this area has still to be explored botanically, particularly from Mount Frederick–Besterskop eastwards along the scarp below The Ruitjies. The relevant localities/properties are summarised as follows (taken from 1:50 000 sheet 3226AD Spring Valley): Finella Falls 1 (parts of this farm were well surveyed in 2009, but there are extensive rocky areas not yet explored); the scarp margins on the Bosch River Spruit 26; Petraea 2 (being the south-western slopes of Mount Frederick and Besterskop); Oribi Fountains 3 (also being the south-eastern slopes of Mount Frederick and Besterskop, as well as the south-facing scarp of The Ruitjies); and those portions of Emerald Hill 26 and adjacent farms that comprise the ‘Groenberg’.

#### Collections and localities.

South Africa, Eastern Cape Province, 3226AD, Farm Emerald Hill 26, Great Winterberg (Adelaide): grassland in road reserve on farm track towards Fenella Falls. 32°22'34"S, 26°20'28"E, 1616 m, 23 January 2009. *Clark VR, Pienaar C, Daniels R 316* (GRA, NBG) (=*Locality 1*).

—Eastern Cape Province, 3226AD, Farm Emerald Hill 26, Great Winterberg (Adelaide): grassland in road reserve on farm track towards Fenella Falls. 32°22'25"S, 26°20'24"E, 1649 m, 6 November 2014. *Clark VR, Bentley L 9* (=*Locality 1; the same population as above, but the 2009 GPS and altitude were a generic reading taken for plant collections along the entire road, and are not as accurate as these provided here. Only photographs were taken of these plants*).

—Eastern Cape Province, 3226AD, Farm Bosch River Spruit 26, Great Winterberg (Adelaide): plateau grassland. 32°23'42"S, 26°21'04"E, 1616 m, 6 November 2014. *Clark VR, Bentley L 11* (GRA) (=*Locality 2; only this plant was collected as a voucher specimen, as the landowner indicated that this area was to be burnt the following day*).

—Eastern Cape Province, 3226AD, Farm Emerald Hill 26, Great Winterberg (Adelaide): moribund grassland on the plateau. 32°23'42"S, 26°21'04"E, 1619 m, 6 November 2014. *Clark VR, Bentley L 12* (=*Locality 3; only photographs were taken of these plants*).

**Figure 1.**
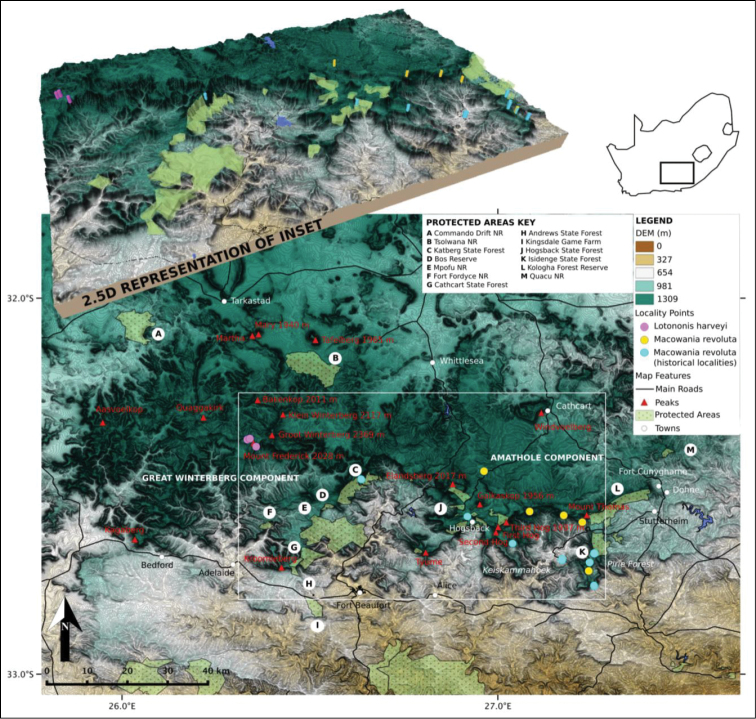
Localities of *Lotononis harveyi* B.-E.van Wyk and *Macowania revoluta* Oliv. in the Great Winterberg–Amatholes, Eastern Cape, South Africa. Cartography by J. Bentley.

### Macowania revoluta

Taxon classificationPlantaeAsteralesAsteraceae

Oliv.

Fig. 1 ; Plate 2

#### Remarks.

*Macowania revoluta*, the type species of *Macowania*, was first collected by Peter MacOwan in the eastern part of the Amatholes sometime prior to 1870 and described by Daniel Oliver in *Icones Plantarum* (Hooker 1867–1871). This almost exclusively southern African genus was later revised by Smith (1927). Relatively few collections of *Macowania revoluta* exist (most specimens being repeat collections by a few historical collectors, see below). Raimondo (2008) indicates that this species had not been re-collected since before 1949, although herbarium investigations by JB indicate that there is one collection from 1976 (albeit with virtually no other data).

The first concrete records of this species’ continued existence was a collection in July 2010 by JB and Nicola Bergh (Compton Herbarium) in the vicinity of Keiskammahoek (Locality 1 – the closest record to the type locality), followed by a second specimen in October 2010 by APD near the Madonna & Child Waterfall in Hogsback (Locality 2). Following this, in December 2014, the species was found by VRC to be abundant in the central Amathole mountains along the Amatola Hiking Trail (Localities 3–5). In March 2015 another plant was recorded by VZ from Isidenge State Forest on the road to Evelyn Hut (one of the overnight huts on the Amatola Hiking Trail; Locality 6).

**Plate 2.**
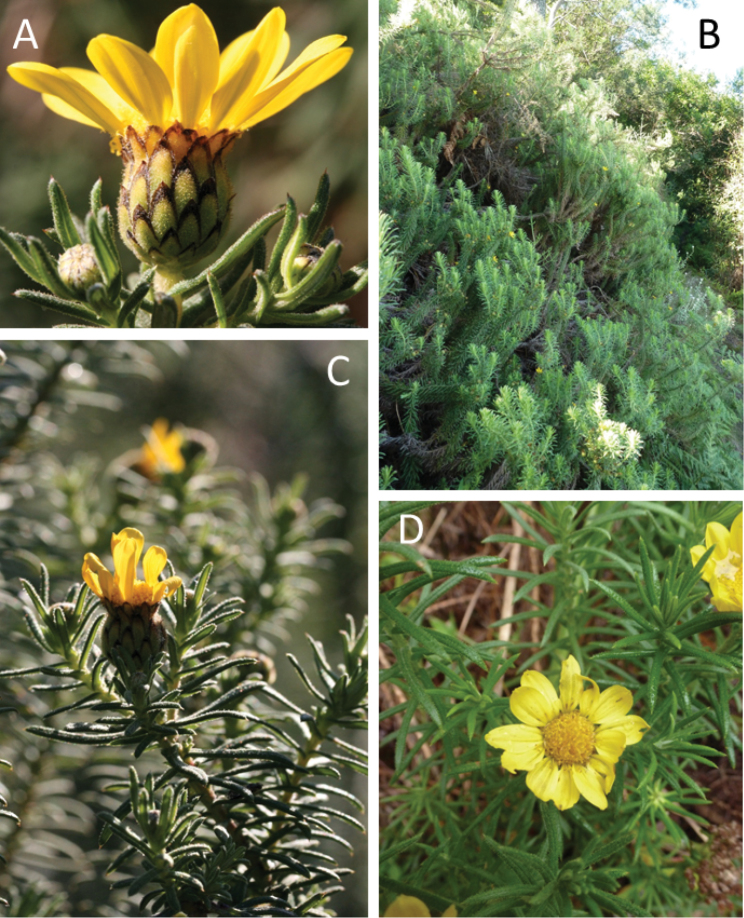
The poorly known Great Winterberg–Amatholes endemic *Macowania revoluta* Oliv. **A** a capitulum showing the distinctive dark involucral bract margins (*Bentley J 1*) **B** shrubby, candelabra growth-habit (above Wolf River Main Forest along the Amathole Hiking Trail, specimen not collected) **C** detail of flowering stem (*Bentley J 1*) **D** young plant showing ruderal tendencies (*Clark VR 451*). Photographs by C. McKune (**A**), V.R. Clark (**B**, **D**) and N. Bergh (**C**).

#### Key characters confirming rediscovery.

The plant is typically an erect, candelabra-like shrub 50 cm to three metres tall, but often lax and weedy when small. The leaves are distinctly linear, dark green, sticky glandular and sweetly aromatic with strongly revolute margins (hence its specific name) and a raised abaxial midrib. Both disc and ray florets are yellow, with the ray floret petals rounded upwards. The involucre is bell-shaped with distinctly long bracts; the margins are strikingly dark-brown.

Another species endemic to the GWA, *Arrowsmithia styphelioides* – earlier believed by Hilliard and Burtt (1985) to be closely affiliated to *Macowania* – has since been found by recent phylogenetic analysis to be nested within *Macowania*, as sister to *Macowania revoluta* (Bentley et al. 2014; the taxonomic revision is currently in progress). *Arrowsmithia styphelioides* differs in its sharply acuminate, ovate leaves, absence of the raised abaxial midrib, as well as in several features of the reproductive organs. Otherwise, no other *Macowania* species are currently known from the GWA (Clark et al. 2014), with the next closest known population of another species (*Macowania pulvinaris* N.E.Br.) being on the Andriesberg, 115 km to the north.

#### Population assessment.

At Locality 1, *Macowania revoluta* was found to be locally abundant, with plants in excess of one meter in height and forming the dominant species. Only one plant was noted at Locality 2, growing on the edges of a derelict *Pinus patula* plantation and *Acacia mearnsii* De Wild. invasions. Locality 3 contained about 20 plants, 0.5–1 m tall, with two in flower and many others in seed. Locality 4 comprised a large colony (ca. 50 m × 100 m in extent) with *Macowania revoluta* (1–3 m tall) forming the dominant species; many were in flower. Locality 5 consisted of a dense but small colony (1–3 m tall) covering ca. 50 m × 10 m; also with many in flower. Only one plant was located at Locality 6, and was not in flower.

#### Habitat and ecology.

Based on the information on the type material, Clark et al. (2014) suggested that this species should be looked for along forest edge and in adjacent grassland. This was a good deduction, as the plants form dense colonies on wet scarp slopes, on cliff-tops, on the margins of indigenous forests, and on the edges of pine plantations and alien thickets. *Macowania revoluta* generally prefers wet areas, and can form the dominant component of mountain fynbos in suitable habitat, co-occurring with various Cyperaceae, *Erica* species, *Pelargonium cordifolium* (Cav.) Curtis, *Psoralea glabra* E.Mey., Pteridium aquilinum (L.) Kuhn subsp. aquilinum, *Rubus rigidus* and *Widdringtonia nodiflora* (L.) Powrie.

#### Conservation status and threats.

*Macowania revoluta* is currently listed as Data Deficient (Raimondo 2008), but is obviously much more common than previously thought. Despite the species’ local abundance, its ruderal tendencies, and being somewhat tolerant of less dense alien vegetation, it is (mostly) known from one quarter degree grid on a small mountain range that is under severe pressure from woody alien invasive species (notably *Acacia dealbata* Link, *Acacia mearnsii*, *Acacia melanoxylon* R.Br. and *Pinus patula*). Furthermore, the potential impacts of climate change on this (and other local montane endemics) is currently unknown. Also, it’s response to fire (and autecology in general) is unknown and requires investigation. Accordingly we recommend the status ‘Rare’.

#### Recommended areas for further exploration.

*Macowania revoluta* potentially occurs anywhere along the wet southern scarp of the Amathole Mountains, between Katberg Pass and Stutterheim. So far it has not been recorded on the adjacent Great Winterberg.

#### Historical collections and localities

**(a selection of these is mapped in Figure 1).** South Africa, Eastern Cape Province, 3227CA & 3227CD, Amathole Mountains (King Williams Town): rocky summit of Pirie Mountain, Buffelsrivier, Kaffraria (label detail differs among the duplicates). October 1887 (this date on the GRA specimen is a bit of an enigma, as it post-dates the species description). *Macowan P 2013* (BOL, E, GH, GRA, K, NYBG, P, PRE; type specimens).

—Eastern Cape Province, 3227CA, Amathole Mountains (King Williams Town): summit of Pirie mountains, Kaffraria. 1200 m (4000'), October 1884. *Leighton (J?) 225* (GRA, NBG, PRE).

—Eastern Cape Province, 3227CA, Amathole Mountains (King Williams Town): summit of Mount Pirie. May 1887. *Tyson W 2935* (PRE).

—Eastern Cape Province, 3227CA, Amathole Mountains (King Williams Town): Perie (=Pirie), Kaffraria. August 1892. *Sim TR s.n.* (BOL).

—Eastern Cape Province, 3227CA, Amathole Mountains (King Williams Town): Pirie. 1200 m (4000'), September 1892. *Sim TR 3283* (NU).

—Eastern Cape Province, 3227CA, Amathole Mountains (King Williams Town): Summit of Perie (=Pirie). 900 m (3000'), November 1893. *Flanagan HG 2144* (GRA, BOL).

—Eastern Cape Province, 3227CA, Amathole Mountains (King Williams Town): Summit of Perie (=Pirie) mountains. 11^th^ September 1901. *Galpin EE 5930* (PRE).

—Eastern Cape Province, 3226DB, Amathole Mountains (Victoria East): Hogsback, common in scrub. January 1920. *Rattray G 304* (GRA).

—Eastern Cape Province, 3227CA, Amathole Mountains (Keiskammahoek): Wolf River Plateau, forest margins in scrub. 29^th^ October 1921. *Stayner FJ 28* (GRA, PRE).

—Eastern Cape Province, 3226DB, Amathole Mountains (Cathcart): Hogsback. September 1925. *Pole Evans IB 1748* (PRE).

—Eastern Cape Province, 3226DB, Amathole Mountains (Stockenstrom): hillside above forest at Hogsback. 28^th^ October 1946. *Esterhuyse E 13,249* (BOL).

—Eastern Cape Province, 3227CA, Amathole Mountains (King Williams Town): Wolf Ridge, Hogsback. 1200 m (4000'), 10^th^ September 1947. *Story R 3119* (GRA, PRE).

—Eastern Cape Province, 3227CA, Amathole Mountains (Keiskammahoek): Wolf River Forest. 18^th^ September 1947. *Dyer RA 104* (GRA).

—Eastern Cape Province, 3227CA, Amathole Mountains (Keiskammahoek): Gwili-Gwili Mountain, old military road to Evelyn Valley. 25^th^ April 1949. *Story R 3797* (PRE).

—Eastern Cape Province, 3227CA, Amathole Mountains (Keiskammahoek): No details. 1976. *Gibbs Russell s.n.* (NU).

—Eastern Cape Province, 3227CA, Amathole Mountains (King Williams Town): summit of Mount Pirie. No date. *Macowan P 9053* (PRE).

—Eastern Cape Province, 3226BC, Katberg (Fort Beaufort): no details. No date. *Macowan P s.n.* (PRE).

—Eastern Cape Province, 3227CA, Amathole Mountains (King Williams Town): Pirie. November, no year. *Sim TR 1029* (PRE).

—Eastern Cape Province, 3227CA, Amathole Mountains (King Williams Town): Pirie. No date. *Sim TR 3130* (NU).

#### Recent collections and localities.

South Africa, Eastern Cape Province, 3227CA, Amathole Mountains (Stutterheim): between isiDengi Forest Station and Evelyn Valley Forestry Station. 32°43'32"S, 27°14'30"E, 1208 m, 27^th^ July 2010. *Bentley J 1 & 5* (NBG) (=*Locality 1*).

—Eastern Cape Province, 3226DB, Amathole Mountains (Cathcart): above Madonna & Child Waterfall, Hogsback, 32°36'27"S, 26°57'47"E, 1106 m, 7^th^ October 2010. *Dold T 15,010* (GRA) (=*Locality 2*).

—Eastern Cape Province, 3227CA, Amathole Mountains (Stutterheim): about one kilometre from Dontsa Hut on the Amatola Hiking Trail (Day 2 from Maden Dam side): in an earth road drain on the edge of a pine plantation next to a forestry road. 32°35'46"S, 27°13'29"E, 948 m, 3^rd^ December 2014. *Clark VR 450* (GRA) (=*Locality 3*).

—Eastern Cape Province, 3227CA, Amathole Mountains (Stutterheim): about five kilometres from Dontsa Hut towards Cata Hut on the Amatola Hiking Trail (Day 3 from Maden Dam side): montane fynbos and streams banks. 32°34'40"S, 27°10'31"E, 1371 m, 4^th^ December 2014. *Clark VR 451* (GRA) (=*Locality 4*).

—Eastern Cape Province, 3227CA, Amathole Mountains (Stutterheim): along the Amatola Hiking Trail towards Zingcuka Hut (Day 5 from Maden Dam side): along a cliff top above Wolf River Main Forest. 32°34'03"S, 27°05'04"E, 1259 m, 6^th^ December 2014 (=*Locality 5; only photographs were taken, by VRC*).

—Eastern Cape Province, 3227CA, Amathole Mountains (Stutterheim): from Isidenge State Forest on the road to Evelyn Hut. 32°43'29"S, 27°14'37"E, 1198 m, 15^th^ April 2015. (=*Locality 6; only photographs were taken, by VZ*).

## Supplementary Material

XML Treatment for Lotononis harveyi

XML Treatment for Macowania revoluta
